# Hospitalization for bronchiolitis in children aged ≤ 1year, Southern Italy, year 2021: need for new preventive strategies?

**DOI:** 10.1186/s13052-023-01455-2

**Published:** 2023-06-06

**Authors:** Maria Elisabetta Baldassarre, Daniela Loconsole, Francesca Centrone, Desiree Caselli, Baldassarre Martire, Lorenzo Quartulli, Angelo Acquafredda, Gabriele D’Amato, Gianfranco Maffei, Giuseppe Latorre, Anita Riganti, Michele Di Noia, Maria Chironna, Nicola Laforgia

**Affiliations:** 1 Unit of Neonatology and Intensive Care, Interdisciplinary Department of Medicine, Aldo Moro University, Bari, 70124 Italy; 2 Hygiene Section, Interdisciplinary Department of Medicine, Aldo Moro University, Bari, 70124 Italy; 3 Pediatric Infectious Diseases Unit, Giovanni XXIII Children Hospital, Azienda Ospedaliero Universitaria Consorziale Policlinico, Bari, 70124 Italy; 4 Unit of Pediatrics and Neonatology, “Monsignor Dimiccoli” Hospital, Barletta, BT Italy; 5 Neonatology Unit, “A. Perrino” Hospital-ASL, Brindisi, Italy; 6 Unit of Pediatrics and Neonatology, “G. Tatarella” Hospital, Cerignola, Foggia Italy; 7 Neonatology Unit, “Di Venere” Hospital – ASL BA, Bari, Italy; 8 Neonatology and Intensive Care Unit, “Ospedali Riuniti” Hospital, Foggia, Italy; 9 Neonatology and Intensive Care Unit, “Miulli” Hospital, Acquaviva delle Fonti, Bari, Italy; 10 Neonatology Unit, “Casa Sollievo della Sofferenza” Hospital, San Giovanni Rotondo, Foggia, Italy; 11 Unit of Pediatrics, “Bonomo” Hospital, Andria, BT Italy

**Keywords:** Bronchiolitis, Respiratory syncytial virus (RSV), Epidemiology, Children, Hospitalization, Intensive care, nirsevimab, Preventive strategies

## Abstract

**Background:**

Bronchiolitis is a major cause of hospitalization in infants, particularly in the first six months of life, with approximately 60–80% of admissions due to respiratory syncytial virus (RSV) infection. Currently, no prophylactic options are available for healthy infants. The present study aimed at describing the demographic, clinical, and epidemiological characteristics of infants hospitalized for bronchiolitis in the Apulia region of Italy in 2021.

**Methods:**

From January to December 2021, data on children aged 0–12 months admitted for bronchiolitis in nine neonatal or pediatric units covering 61% of pediatric beds of hospitals in the Apulia region of Italy were analyzed. Demographic data, comorbidities, need for oxygen support, length of hospital stay, palivizumab administration, and outcomes were collected. For the purpose of the analysis, patients were divided into those aged 0–3 months and > 3 months. A multivariate logistic regression model was used to explore associations between the need for oxygen support and sex, age, comorbidities, history of prematurity, length of hospital stay, and palivizumab administration.

**Results:**

This study included 349 children aged 0–12 months admitted for bronchiolitis, with a peak of hospitalization in November (7.4 cases/1,000 children). Of these patients, 70.5% were RSV positive, 80.2% were aged 0–3 months, and 73.1% required oxygen support. Moreover, 34.9% required observation in the sub-intensive care unit, and 12.9% in the intensive care unit. Of the infants who required intensive care, 96.9% were aged 0–3 months and 78.8% were born at term. Three patients required mechanical ventilation and one, who required Extra Corporeal Membrane Oxygenation, died. Children aged 0–3 months were more likely to show dyspnea, need oxygen support, and have a longer hospital stay.

**Conclusions:**

The present study showed that almost all of the children who required intensive care support were aged ≤ 3 months and most were born at term. Therefore, this age group remains the highest risk group for severe bronchiolitis. Preventive measures such as single-dose monoclonal antibody immunoprophylaxis, and maternal and childhood vaccination against RSV, may reduce the high public health burden of bronchiolitis.

## Background

Bronchiolitis is a leading cause of hospital admission for infants and young children worldwide, accounting for 15–17% of all hospitalizations in children aged < 2 years [1, 2]. Children with bronchiolitis typically present with signs of respiratory distress and acute lower respiratory tract infections (ALRTIs) [1]. Respiratory syncytial virus (RSV) is responsible for 60–80% of bronchiolitis cases in infants and could lead to severe disease and death in children aged < 5 years, especially during the first 6 months of life [1, 3–5]. It has been estimated that almost all children have been infected with RSV by age 2 years [6, 7].

Globally, RSV infection is responsible for 3.1 million episodes of RSV-ALRTIs, resulting in about 3.2 million hospital admissions and 59,600 in-hospital deaths among children aged < 5 years, making RSV-ALRTIs the third leading cause of death in this age group [5, 8]. Hospitalization for RSV in young children has been reported to peak during the first 12 months of life, with 45% of hospital admissions and in-hospital deaths due to RSV-ALRTIs occurring in children aged < 6 months [5, 9].

In temperate regions, bronchiolitis shows a seasonal pattern, characterized by an increase in number in late October, a peak in January/February, and an ending in April [10]. In 2021, the COVID-19 pandemic affected the rate of infant hospitalization for acute bronchiolitis, with a drastic reduction in number of patients during in the usual seasonal peak period [11]. Unexpectedly, many countries experienced an interseasonal resurgence of RSV infections in late 2021 [12–14]. In Italy, RSV infection had almost disappeared during the 2020–2021 season [15–17].

Yearly, hospital pediatric wards must deal with large numbers of children, especially infants, with bronchiolitis. The hospitalization cost for acute bronchiolitis in Europe is about 2,000 euros per patient in pediatric wards and 8,000 euros per patient in pediatric intensive care units, with the costs of hospitalization being significantly higher for RSV bronchiolitis than for bronchiolitis caused by other etiologies [18, 19]. Moreover, the costs of hospitalization for bronchiolitis have been reported to be higher for children aged ≤ 3 months than for older children [19].

Supportive therapy rather than interventional therapy, has been recently recommended for patients with acute viral bronchiolitis [1]. Because no specific therapies are available, preventive measures represent the only chance to reduce the burden of such infections. Prophylaxis with the monoclonal antibody palivizumab may reduce the severity of RSV associated bronchiolitis and hospitalizations for RSV infection. To date, however, palivizumab has been recommended only for infants born at gestational age ≤ 35 weeks and aged < 6 months at the beginning of the RSV season, and for children aged < 2 years with major risk factors, requiring up to five monthly administrations throughout the RSV season [20, 21]. Currently, no prophylaxis options are available for healthy term and preterm infants. New preventive strategies, such as the single-dose monoclonal antibody nirsevimab, and maternal and childhood vaccines, are about to be developed [1].

The present study aimed at describing the clinical and epidemiological characteristics of infants aged 0–12 months hospitalized with a diagnosis of bronchiolitis in neonatal or pediatric units of the Apulia region (Southern Italy) during in the 2021 out-of-season epidemic.

## Methods

Data on children aged 0–12 months in the Apulia region of Italy, which contains about 4 million people, who were hospitalized for bronchiolitis from January to December 2021 were collected and analyzed. Nine neonatal or pediatric units covering 61% pediatric beds of hospitals in Apulia were involved in the study, with these units providing retrospectively collected data. Eligible patients were enrolled by searching discharge reports for the following diagnoses, coded according to the International Classification of Diseases, Ninth Revision, Clinical Modification (ICD-9-CM): 079.6 (RSV infection), 466.1 (acute bronchiolitis), 466.11 (acute bronchiolitis due to RSV), and 466.19 (acute bronchiolitis due to pathogens other than RSV). RSV infection was ascertained through the use of molecular tests (real-time PCR). Moreover, at admission, all children were tested for SARS-CoV-2 infection through an antigenic or molecular test (real-time PCR). Demographic data, comorbidities, need for oxygen support, length of hospital stay, palivizumab administration, and outcomes were collected by filling in a file with coded variables for each patient. Data were analyzed using STATA 14.0 software (StataCorpLLC, College Station, TX, USA). Patients were divided into those aged 0–3 months and > 3 months. Proportions were compared by chi-squared tests, whereas the average numbers of days from symptom onset to hospitalization and the average length of hospital stay in the two groups were compared by t-tests. A p-value ≤ 0.05 was considered statistically significant. A multivariate logistic regression model was used to explore associations between the need for oxygen support and sex, age, comorbidities, history of prematurity, length of hospital stay, and palivizumab administration.

All procedures performed in the study were in accordance with the ethical standards and the Declaration of Helsinki, as revised in 2013. Ethical approval was obtained from the Institutional Review Board at the Apulian Regional Observatory for Epidemiology (n. 614|04 of 4 January 2022). Informed consent was waived because all data were deidentified.

## Results

Between 1 January and 31 December 2021, 349 children aged 0–12 months were hospitalized for bronchiolitis in nine neonatal or pediatric units in the Apulia region of Italy. Of these patients, 280 (80.2%) were aged 0–3 months. Etiologically, bronchiolitis was attributed to RSV infection in 246 (70.5%) patients. For the remaining 29.5% of cases, molecular testing for RSV detection was not performed and the diagnosis was based on clinical data. The demographic and clinical characteristics of all patients are summarized in Table 1.

**Table 1:** Demographic and clinical characteristics of the 349 children aged 0–12 months in the Apulia region of Italy hospitalized for bronchiolitis in 2021

	N	%
Total	349	
Sex		
Male	193	55.3%
Female	156	44.7%
Median age, months (IQR)	1 (0–2)	
Age groups (months)		
0–3	280	80.2%
4–6	40	11.5%
7–9	22	6.3%
10–12	7	2.0%
Prematurity at birth	48	13.8%
Comorbidity	42	12.0%
Need for oxygen support	255	73.1%
Symptoms		
Fever	76	21.8%
Dyspnea	309	88.5%
Feeding difficulties	172	49.3%

**Fig. 1:**
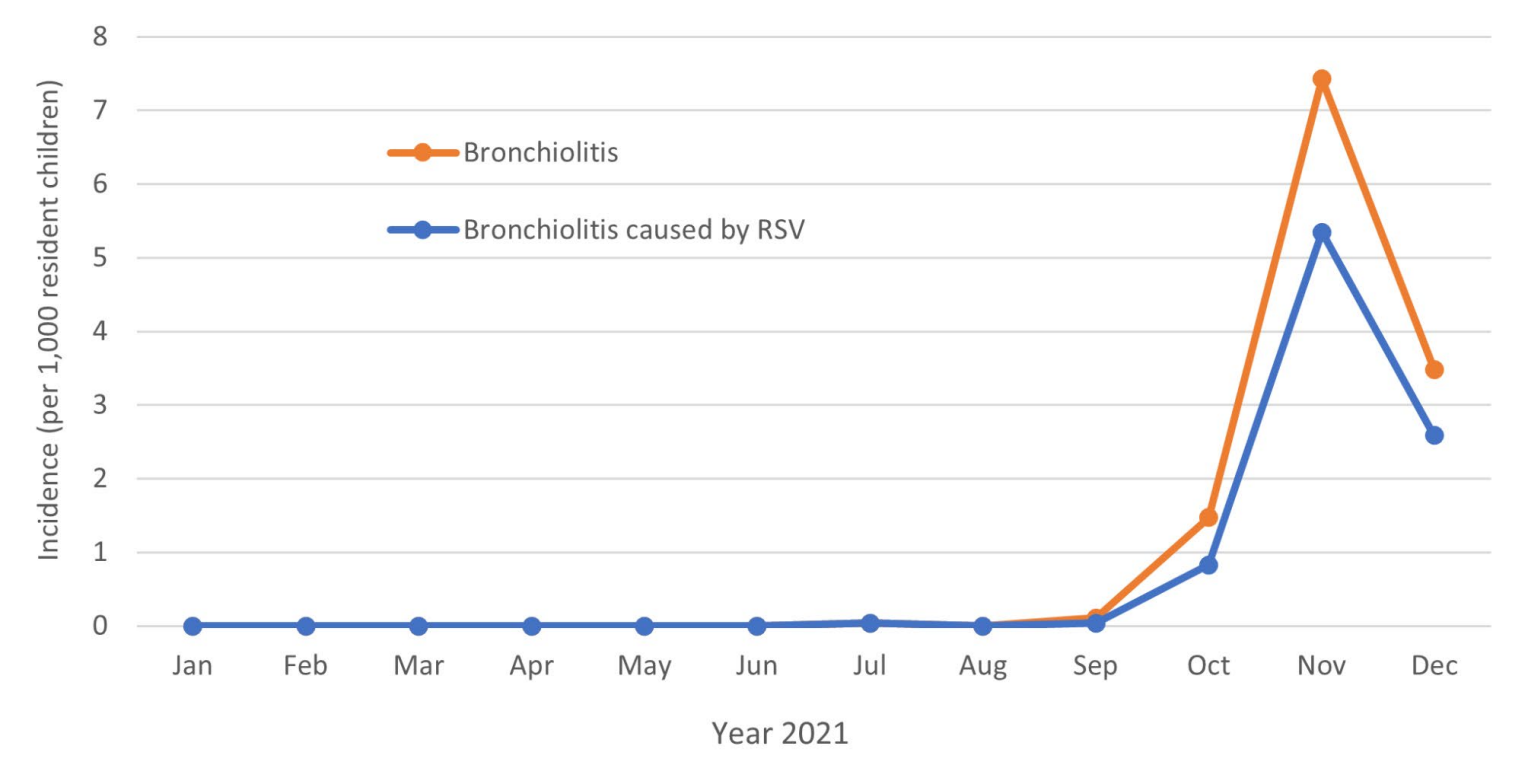
Incidence rates (per 1,000 children aged 0–12 months) of all bronchiolitis and bronchiolitis tested positive for RSV, by month of hospitalization, Apulia region, year 2021

Overall, 255 (73.1%) of the 349 patients required additional oxygen support; of these, 89/255 (34.9%) required observation in the neonatal sub-intensive care unit, and 33/255 (12.9%) in the neonatal intensive care unit. In particular, of the 33 patients in the neonatal intensive care unit, 19 (57.6%) required nasal continuous positive airway pressure (n-CPAP), three (9.0%) required nasal intermittent positive pressure ventilation (n-IPPV), and three (9.0%) required endotracheal intubation and mechanical ventilation. One patient aged one month with severe comorbidities required extracorporeal membrane oxygenation (ECMO). All the hospitalized children were tested for SARS-CoV-2 infection at admission. Only 4 children resulted positive for SARS-CoV-2 infection. All of them were aged 0–3 months and none required intensive care support. Overall, only 13 (3.7%) patients received at least one dose of palivizumab. The average time between the onset of symptoms and hospitalization was 3.7 days (range: 0–45 days), and the average length of hospital stay was 7.1 days (range: 1–76 days). The patient who required ECMO died. The incidence rates (per 1,000 resident children aged 0–12 months) of all bronchiolitis and bronchiolitis tested positive for RSV, by month of hospitalization, are shown in Fig. 1. Hospitalizations for bronchiolitis and RSV-caused bronchiolitis peaked in November 2021 (n = 207) with a incidence rate of 7.4 cases/1,000 children.

Of the 33 babies who needed intensive care support, 32 (96.9%) were aged 0–3 months and 26 (78.8%) were born at term. Table 2 compared the demographic and clinical characteristics of patients aged 0–3 months and > 3 months.

**Table 2:** Demographic and clinical characteristics of patients aged 0–3 months and > 3 months with bronchiolitis in the Apulia region, year 2021

	0–3 months age group			> 3 months age group		*p-value*
	N	%		N	%	
Total	280	80.2%		69	19.8%	
Sex						
Male	159	56.8%		34	49.3%	0.261
Female	121	43.2%		35	50.7%	
Prematurity at birth	35	12.5%		13	18.8%	0.209
Comorbidity	25	8.9%		17	24.6%	< 0.001
Oxygen support	219	78.2%		36	52.2%	< 0.001
Symptoms						
Fever	49	17.5%		27	39.1%	< 0.001
Dyspnea	254	90.7%		55	79.7%	0.01
Feeding difficulties	140	50.0%		32	46.4%	0.59
Time from symptom onset to hospitalization (mean, days)	3.2			5.7		< 0.001
Length of hospital stay (mean, days)	7.7			4.9		< 0.001

Children aged > 3 months were significantly more likely to present with comorbidities and fever (p < 0.001 each), whereas children aged 0–3 months were significantly more likely to present with dyspnea (p = 0.01) and need for oxygen support (p < 0.001). Moreover, the number of days from symptom onset to hospitalization was significantly lower (p < 0.001) and the length of hospital stay significantly higher (p < 0.001) in children aged 0–3 months than in those aged > 3 months. Multivariate logistic regression revealed that age (OR: 0.79, 95% CI: 0.71–0.88), comorbidities (OR: 4.5, 95% CI: 1.43–14.22), and length of hospital stay (OR: 1.2, 95% CI: 1.11–1.32) were significantly related to the need for oxygen support.

## Discussion

Viral bronchiolitis is a common cause of hospitalization in young children. Despite RSV being recognized as the main cause of bronchiolitis presentations in infants, the community burden of RSV infections still remains underestimated worldwide. Severe bronchiolitis mainly affects children aged < 6 months [3–5]. However, the COVID-19 pandemic altered the epidemiology and seasonality of many respiratory viruses, causing out-of-seasonal outbreaks worldwide [22].

The present study analyzed children aged 0–12 months in the Apulia region of southern Italy who were hospitalized for bronchiolitis in 2021. Most of these children were aged ≤ 3 months, with no differences between boys and girls. Four children aged 0–3 months showed a coinfection with SARS-CoV-2 but none of them required intensive care support, suggesting that the severe clinical picture of children who required intensive care support was not associated with COVID-19. A single center study in the Apulia region found that younger children were more likely to present with dyspnea and require oxygen support, whereas older children were more likely to present with fever and comorbidities [17]. Despite the low prevalence of risk factors, more than 70% of these children required oxygen support. In particular, only 13% of these children were born prematurely, rates lower than in recent studies from Italy (24.5%) and the USA (33%) [23, 24]. Only 12% of the children in the present study had comorbidities, similar to the percentage in a Norwegian study but lower than that reported in children from the USA [24, 25].

It has been demonstrated that most children hospitalized with RSV infection were previously healthy, with only 3% of RSV-infected children in primary care having comorbidities and only 5% born prematurely [26, 27]. These findings indicate that targeting preventive strategies exclusively at high-risk children would have a limited effect on the total disease burden of RSV infection. It has been estimated that 93% of infants hospitalized were ineligible for prophylaxis with palivizumab [28]. The low prevalence of risk factors in this population, making them ineligible for prophylaxis, suggests that an all-infant immunization strategy should be considered after a cost-effectiveness analysis.

Acute seasonal bronchiolitis is an important public health issue, with severe RSV disease having significant economic costs for healthcare systems. RSV was found to reduce health‑related quality of life (HRQoL) almost 40% during the first week after onset of symptoms in patients aged < 2 years, when the illness is most severe [29]. The disease also has an impact on parents/caregivers, as they experience worry, anxiety and distress [29]. A recent Italian study estimated that the mean cost of hospitalization for bronchiolitis in a pediatric ward was more than 5,000 euros, with the cost being higher for hospitalization in pediatric intensive care units (PICU) [19]. Nearly 35% of the children in the present study who required oxygen support needed to stay in neonatal sub-intensive care units, as they needed high flow oxygen administration. Moreover about 13% of patients requiring oxygen administration needed intensive care support (n-CPAP, n-IPPV, endotracheal intubation), with almost 80% being full-term newborns. A previous European study estimated that the average cost of PICU hospitalization for bronchiolitis in children aged < 12 months was four times higher than the cost for hospitalization in a pediatric ward and more than 20 times higher than children managed in the Emergency Department [30]. More than 80% of the children described in the present study were aged 0–3 months. This age range has been reported to be a risk factor for severe bronchiolitis, with hospital admission for bronchiolitis in this age group associated with higher costs [19, 31]. Because almost all the children in the present study requiring hospitalization in the PICU were aged 0–3 months, preventive strategies aimed at protecting this age group, such as maternal vaccination and monoclonal antibody immunoprophylaxis for newborns, could potentially reduce the economic impact of bronchiolitis [19].

Currently, the only approved prophylactic measure is the administration of palivizumab. Palivizumab, however, is expensive, provides short-lived protection, and re-quires up to five monthly doses [32, 33]. The approval of the new long-acting mono-clonal antibody nirsevimab is the next expected advance in RSV prevention. In term and preterm infants, nirsevimab showed an overall efficacy of 75% in preventing ALRTIs requiring medical attention [28]. Moreover, when compared with placebo, nirsevimab showed greater ability to prevent hospitalization and provided a longer duration of protection [34]. Use of this preventive strategy in all infants during their first RSV season has been estimated to reduce direct medical costs 49%, although the costs of nirsevimab could represent a barrier to its implementation [28, 35]. A recent evaluation of the cost-effectiveness of nirsevimab programs in England and Wales revealed that, at a price per dose of £63, a large-scale seasonal program would be a cost-effective strategy for all infants at birth [33]. Moreover, by reducing the numbers of patients infected with RSV, this strategy would reduce the numbers of patients prescribed antibiotics, reducing both costs and the negative effects of antimicrobial resistance on other pathogens [33]. Despite evidence suggesting that antibiotics do not benefit patients with viral bronchiolitis, due to the low rate of bacterial superinfections, the use of antibiotics is still widespread [1, 24, 36].

The study had several limitations. First, data on the etiology of bronchiolitis were available for only 70% of the included patients, with the remaining 30% not undergoing molecular tests for RSV. The recent proposal of the World Health Organization to add RSV testing to existing influenza surveillance systems could lead to the implementation of molecular testing, at least in hospital settings [37, 38]. Second, data on possible coinfection with viruses other than SARS-CoV-2 were not available. The presence of coinfections could have affected length of hospital stay or the need for oxygen support in some patients with severe bronchiolitis.

## Conclusions

The clinical and economic burden of viral bronchiolitis is still underestimated. Currently, RSV disease affects millions of children every year and is associated with high costs, even for patients with mild disease. The present study confirmed that children aged ≤ 3 months remain the highest risk group for severe bronchiolitis and admission to intensive care units. Preventive measures such as single-dose monoclonal antibody immunoprophylaxis, and maternal and childhood vaccination against RSV may reduce the high public health burden of bronchiolitis.

## Data Availability

Data are available on request from the corresponding author.

## List of abbreviations

RSV: respiratory syncytial virus
ALRTIs: acute lower respiratory tract infections
ICD-9-CM: International Classification of Diseases, Ninth Revision, Clinical Modification
n-CPAP: nasal continuous positive airway pressure
n-IPPV: nasal intermittent positive pressure ventilation
ECMO: extracorporeal membrane oxygenation
HRQoL: health‑related quality of life
PICU: pediatric intensive care units
